# Revisiting the mechanisms of mid-Tertiary uplift of the NE Tibetan Plateau

**DOI:** 10.1093/nsr/nwad008

**Published:** 2023-01-09

**Authors:** Hong-Hong Wei, Guo-Li Wu, Lin Ding, Long-Gang Fan, Lin Li, Qing-Ren Meng

**Affiliations:** State Key Laboratory of Tibetan Plateau Earth System, Environment and Resources (TPESER), Institute of Tibetan Plateau Research, Chinese Academy of Sciences, Beijing 100101, China; Institute of Geomechanics, Chinese Academy of Geological Sciences, Beijing 100081, China; State Key Laboratory of Tibetan Plateau Earth System, Environment and Resources (TPESER), Institute of Tibetan Plateau Research, Chinese Academy of Sciences, Beijing 100101, China; State Key Laboratory of Lithospheric Evolution, Institute of Geology and Geophysics, Chinese Academy of Sciences, Beijing 100029, China; Department of Geosciences, University of Arizona, Tucson, AZ 85716, USA; State Key Laboratory of Lithospheric Evolution, Institute of Geology and Geophysics, Chinese Academy of Sciences, Beijing 100029, China; University of Chinese Academy of Sciences, Beijing 100049, China

**Keywords:** NE Tibetan Plateau, mid-Tertiary, unconformity, volcanism, edge convection

## Abstract

Contrasting views exist on timing and mechanisms of Tertiary crustal uplift in the NE Tibetan Plateau based on different approaches, with many models attributing surface uplift to crustal shortening. We carry out a comprehensive investigation of mid-Tertiary stratigraphy, sedimentology, and volcanism in the West Qinling, Hoh Xil and Qaidam basin, and the results challenge previous views. It was held that the discordance between Oligocene and Miocene strata is an angular unconformity in the West Qinling, but our field observations show that it is actually a disconformity, indicative of vertical crustal uplifting rather than crustal shortening at the Oligocene to Miocene transition. Widespread occurrence of synsedimentary normal faults in mid-Tertiary successions implicates supracrustal stretching. Miocene potassic–ultrapassic and mafic–ultramafic volcanics in the Hoh Xil and West Qinling suggest a crucial role of deep thermomechanical processes in generating crust- and mantle-sourced magmatism. Also noticeable are the continuity of mid-Tertiary successions and absence of volcanics in the Qaidam basin. Based on a holistic assessment of stratigraphic–sedimentary processes, volcanic petrogenesis, and spatial variations of lithospheric thicknesses, we speculate that small-sale mantle convection might have been operating beneath northeast Tibet in the mid-Tertiary. It is assumed that northward asthenospheric flow was impeded by thicker cratonic lithosphere of the Qaidam and Alxa blocks, thereby leading to edge convection. The edge-driven convection could bring about surface uplift, induce supracrustal stretching, and trigger vigorous volcanism in the Hoh Xil and West Qinling in the mid-Tertiary period. This mechanism satisfactorily explains many key geologic phenomena that are hardly reconciled by previous models.

## Teaser

Mid-Tertiary uplift of the NE Tibetan plateau is likely driven by edge convection, and this mechanism explains a wide range of prominent phenomena such as regional surface vertical motion, supracrustal extension, generation/distribution of coeval volcanism, and Late Miocene switch of tectonic regimes.

## INTRODUCTION

The collision of India with Asia occurred around 55–60 Ma and built the Tibetan Plateau [1–4]. It, however, remains controversial how the plateau expanded laterally and rose in response to the persistent India–Asia convergence [4,5]. Diverse dynamic models have been advanced to reconstruct the spatiotemporal growth of the Tibetan Plateau [6–9]. Some workers suggest that the NE Tibetan Plateau began developing soon after the India–Asia collision [10], but others insist that the Tibetan Plateau expanded outward in steps, with its peripheral margins not having been involved until the Miocene [11]. No agreement has been reached as to how the NE Tibetan Plateau evolved. A great many studies have been carried out to understand different aspects of tectonic history of NE Tibet, such as stratigraphic and sedimentary evolution [12–15], basin tectonics [16–18], exhumation history of structural belts [19–23], petrogenesis of volcanic rocks [24–28], crustal deformation [10,29,30], and crustal and lithospheric structures [31–34].

This work focuses on mid-Tertiary tectonics of the NE Tibetan Plateau based mainly on stratigraphic and sedimentologic observations in the West Qinling, Hoh Xil and Qaidam basin. Our revisit of stratigraphic sequences and sedimentary processes reveals that the West Qinling and Hoh Xil orogens underwent crustal vertical uplifting rather than shortening in the mid-Tertiary, and surface uplift was accompanied by volcanism sourced from both the crust and mantle. The Qaidam basin, by contrast, is free of volcanism and characterized by conformable mid-Tertiary sequences. Based on detailed investigations of stratigraphic and sedimentary successions, together with careful evaluation of petrogenesis of volcanic rocks and spatial variations of the lithospheric thickness, we assume that an edge-driven convection might have been operating beneath the NE Tibetan Plateau in the mid-Tertiary as a consequence of impediment of the thicker lithospheric keels of the Qaidam and North China cratonic blocks to northward asthenospheric flow. The resultant upwelling of the asthenospheric hot materials raised the crustal surface and simultaneously triggered volcanism. This mechanism provides a satisfactory explanation for a number of prominent geologic phenomena such as regional disconformity, supracrustal stretching, and spatial distribution of the crust- and mantle-sourced volcanism.

## GEOLOGIC SETTING

**Figure 1.**
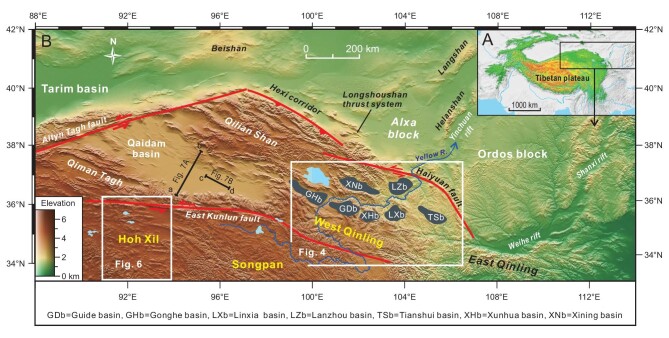
(A and B) Geomorphologic map showing distribution of main tectonic elements and Cenozoic intermontane basins in the NE Tibetan Plateau.

The NE Tibetan Plateau is made up of both structural/orogenic belts and cratonic blocks (Fig. 1). The western portion comprises the Qaidam basin and surrounding structural belts such as the Altyn Tagh, Qilian Shan, East Kunlun and Hoh Xil, whereas the eastern portion is a mosaic of intermontane basins and structural belts. The eastern portion is largely occupied by the West Qinling, a wide orogenic domain that experienced multiple terrane accretion, magmatism, and metamorphism in the Paleozoic and early Mesozoic [35–38]. The West Qinling was further modified by the alternating crustal extension and contraction in the late Mesozoic [17].

**Figure 2.**
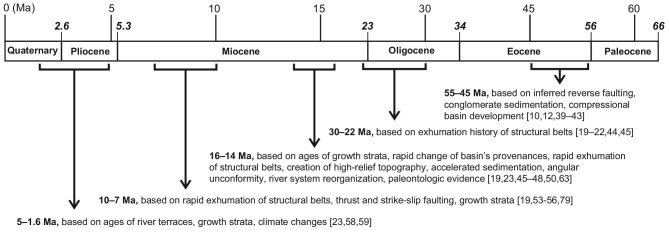
A summary of the views on timing of uplift of the NE Tibetan Plateau. Diverse approaches are used to infer the ages of contraction-induced uplift, and tectonic interpretations are based on different rationales of geological phenomena and experimental data.

Opinions differ considerably on when and how the NE Tibetan Plateau was established (Fig. 2). Surface uplift of NE Tibet is commonly attributed to crustal shortening and thickening due to far-field effect of the India–Asia collision. Crustal contraction is assumed to have been initiated as early as the Early Eocene on account of initiation of reverse faulting [39,40], compressional basins [12,17,41] and conglomeratic sedimentation [10,42,43]. Low-temperature thermochronologic data show that structural belts in NE Tibet underwent cooling/rock exhumation in the interval from ∼30 to 22 Ma, which is often ascribed to crustal shortening [19–22,44,45]. The inference of the ∼30–22 Ma shortening event, however, is not justified because of the lacking of geologic evidence of simultaneous thrust faults and growth strata. By contrast, ∼16–14 Ma crustal shortening and surface uplifting are well confirmed by a variety of geological observations such as growth strata [46], thrust and strike-slip faulting [19,47], marked increase in sedimentation rates [48,49], river system reorganization [50], and rapid exhumation of structural belts and coeval shifting of sediment supply systems of adjacent basins [23,49,51,52]. Rapid contraction-related exhumation of structural belts is demonstrated to occur at ∼10–8 Ma by virtue of growth strata [53,54], cooling ages [55,56] and transpressional deformations [57]. The Pliocene witnesses another period of significant surface uplift that is recorded by rapid fluvial incision, rock denudation and high-rate sedimentation of coarse-grained sediments [23,58–60]. Figure 2 summarizes the key time intervals when the NE Tibetan Plateau is inferred to experience rapid uplift in response to crustal shortening.

Vigorous debate exists regarding when crustal shortening initiated in the NE Tibetan Plateau. Paleogene successions of intermontane basins in the West Qinling, such as the Xining, Guide and Linxia basins, are interpreted as infill of flexural or compressional basins [17,41,61]. The Eocene–Oligocene Lulehe conglomerate in the western and middle Qaidam basin is also taken as sedimentary record of thrust-related uplifting of the Qilian Shan in the north [10,42,43]. Dating of some reverse faults hints at Eocene inception of shortening in the West Qinling [39]. All these studies suggest Eocene–Oligocene contractional deformations, implying an immediate response of NE Tibet to the India–Asia collision. Recent structural and stratigraphic investigations, however, show that the eastern NE Tibetan Plateau was in an extensional setting during the Paleogene on account of widespread occurrence of normal faulting that either controls Paleogene sedimentation or occur in Paleogene successions [62]. Early Miocene tectonic setting also remains elusive albeit ∼30–22 Ma exhumation of some structural belts, such as the East Kunlun and Laji Shan, is often interpreted as the result of crustal shortening [20–22,44,45,51,63]. Middle–Late Miocene rock uplift, thrusting and strike-slip faulting have been widely documented throughout the NE Tibetan Plateau [19,49,53,64,65], and it is now accepted that crustal shortening had become predominant since ∼15 Ma [46,48,53].

## STRATIGRAPHIC SEQUENCES

**Figure 3.**
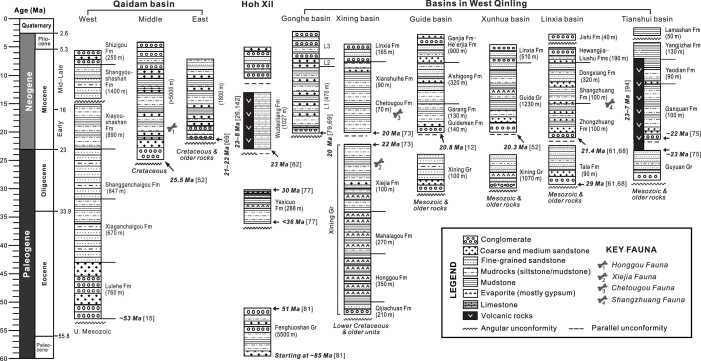
Tertiary stratigraphy of the NE Tibetan Plateau. Chronostratigraphic framework is based on mammal fossils and magnetostratigraphic data. Note the prominent unconformity separating the Miocene from the underlying units. Also noteworthy are Miocene volcanic rocks in the Hoh Xil and West Qinling as well as their absence in the Qaidam basin. The number in parenthesis represents the referenced paper.

Tertiary strata in the NE Tibetan Plateau have been well studied, and the age assignments to lithostratigraphic units rely basically on mammal fossils and magnetostratigraphy [12–15,52,61,66–71]. Tertiary successions are perfectly preserved in many basins, and rest unconformably on Mesozoic and older rocks (Fig. 3).

### The West Qinling

**Figure 4.**
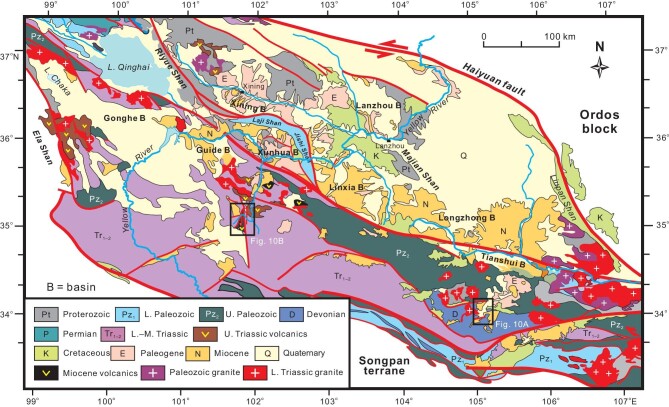
Simplified geological map showing distribution of Cenozoic intermontane basins in the West Qinling. Note that original configurations of Paleogene and Early Miocene basins were altered to various degrees by late-stage crustal deformations.

**Figure 5.**
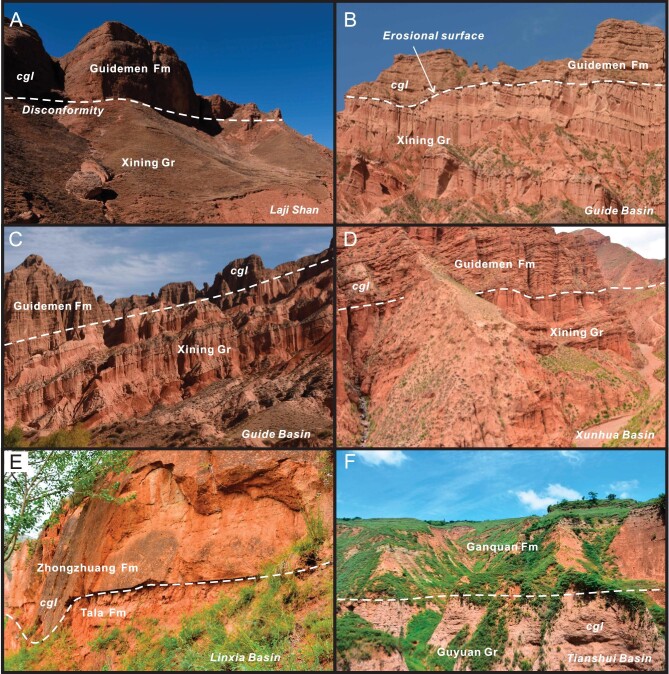
Field photos showing disconformities between Paleogene and Miocene strata in the West Qinling. (A) Disconformable surface between the Guidemen conglomerate and the underlying mudrocks of the Xining Group in the Laji Shan. Width of view ∼200 m. (B) An erosional low-angle unconformity resulting from fluvial channeling into the Xining Group in the Guide basin. Width of view ∼250 m. (C) Typical disconformity separating the Guidemen conglomerate from the underlying Xining Group in the Guide basin. Width of view ∼150 m. (D) Sharp discordance (disconformity) between the Guidemen Formation conglomerate and the Xining Group mudrocks in the Xunhua basin. Width of view ∼300 m. (E) A sharp erosional contact (disconformity) between basal conglomerate of Miocene Zhongzhuang Formation and Oligocene Tala Formation in the Linxia basin. Width of view ∼10 m. (F) Sharp contact (disconformity) between lacustrine mudrocks of the Lower Miocene Ganquan Formation and alluvial conglomerate of the Oligocene Guyuan Group in the Tianshui basin. Width of view ∼150 m. Fm = Formation, Gr = Group, cgl = conglomerate.

A number of Cenozoic intermontane basins exist in the West Qinling (Figs 1 and 4). A regional unconformity separates Paleogene from Neogene strata (Fig. 3) and can be readily observed in the field (Fig. 5). Tertiary sequence in the Xining basin was previously regarded conformable [66,72], but a careful magnetostratigraphic study identifies a disconformity separating the Xining Group from overlying Miocene Chetougou Formation, which represents a time lapse from 22 to 20 Ma [73]. This disconformity becomes conspicuous in the Laji Shan (Fig. 5A), which bounds the Xining basin on the south and was once a part of the Xining basin in the Paleogene [62]. The unconformity between the Paleogene and Miocene in the Guide basin is often taken as an angular unconformity [41]. However, this low-angle discordance turns out to be a local erosional surface in that Lower Miocene Guidemen Formation remains in general parallel to the underlying Xining Group (Fig. 5B). Field observations further disprove the interpretation of angular unconformity insofar as the ‘angular contact’ changes laterally into a disconformity in a short distance (Fig. 5C). The erosional contact resulted from channeling of rivers that deposited basal conglomerate of the Guidemen Formation. This disconformity is also clearly discerned in the Xunhua and Linxia basins where it manifests itself as a striking erosional surface below the Guidemen and Zhongzhuang formations (Fig. 5D and E), with paleosols being locally present atop the Upper Oligocene Tala Formation [74]. The coeval disconformity in the Tianshui basin is exemplified by a sharp facies change from the Guyuan fluvial conglomerate to Ganquan lacustrine fine-grained clastic rocks (Fig. 5F) [75]. Paleogene strata are missing in the Gonghe basin where Miocene units rest directly on strata of different ages [76].

### The Hoh Xil

**Figure 6.**
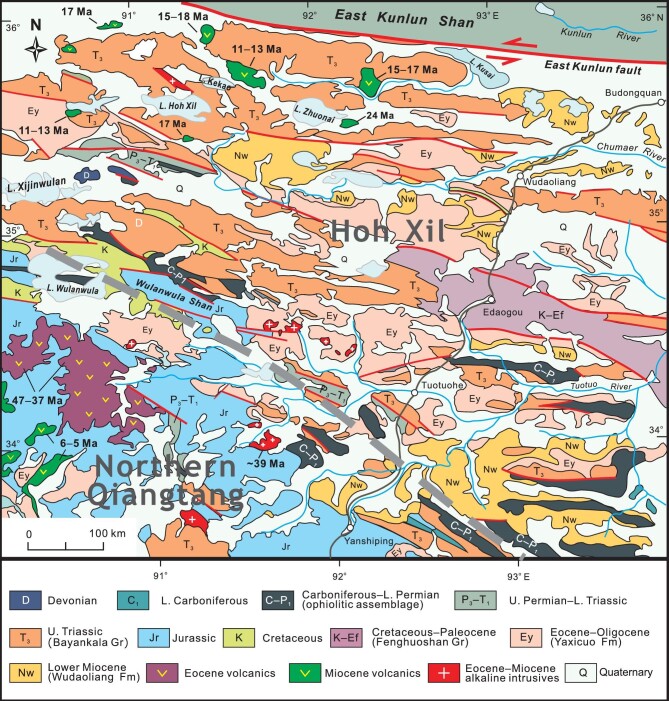
Simplified geologic map showing distribution of Tertiary strata and volcanic rocks in the Hoh Xil and northern Qiangtang. Note that Lower Miocene Wudaoliang Formation (Nw) unconformably overlies the pre-Miocene strata. The dates of Miocene volcanic rocks are from Refs [24,26,28].

Tertiary sequence in the Hoh Xil is composed of three units, the Fenghuoshan Group, Yaxicuo Formation and Wudaoliang Formation in ascending order, and two unconformities separate them from each other (Fig. 3). The Late Eocene–Early Oligocene Yaxicuo Formation is widely distributed (Fig. 6) and dated at 36–30 Ma by magnetostratigraphic studies [77]. This unit is deformed to various degrees and particularly intensely folded near the Tanggula, Fenghuoshan and Hoh Xil thrust belts [29,78,79]. In contrast, the overlying Wudaoliang Formation remains flat-lying throughout the Hoh Xil, indicative of termination of crustal shortening since the Miocene [80]. Thrusting and folding should have come to an end by the Late Oligocene inasmuch as the deformed Yaxicuo Formation is overlain by the undeformed basalt sheets dated at ∼27 Ma [79,81]. The Wudaoliang Formation is assigned to be a Lower–Middle Miocene unit from 23 to 16 Ma on the basis of magnetostratigraphic dating and fossil assemblages [82,83]. The discordance between the Yaxicuo and Wudaoliang formations mostly expresses itself as an angular unconformity [29,78,84] although it is observed as a disconformity in some locales [80].

### The Qaidam basin

**Figure 7.**
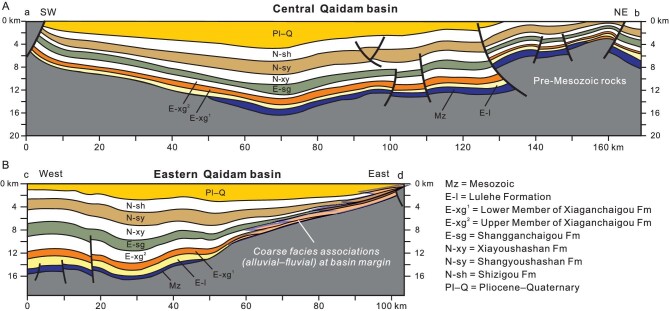
Geological sections of the Qaidam basin based on interpretations of seismic profiles. (A) SW–NE-striking section across the central part of the basin, showing the continuity of Cenozoic successions. Basinward thrusting had not started until the Late Miocene when the Shizigou Formation was deposited as growth strata. (B) The west–east-striking section in the eastern Qaidam basin, showing transgressive overlapping of units toward the east. Refer to Fig. 1 for locations of the two sections.

Cheng *et al*. [16] provide a helpful review of Cenozoic stratigraphy of the Qaidam basin, and make a discussion about the controversy on age assignments to lithostratigraphic units in the middle of the basin [14,52]. Figure 7A displays a geologic section across the central Qaidam basin, showing the continuity of Cenozoic successions. Given that all the Tertiary successions of the basin were deposited in continental environments, sedimentary facies associations may exhibit marked spatial variations, with some units being diachronous. As a result, simple temporal correlation of lithostratigraphic units can be misleading. For example, the Lulehe Formation is dated or inferred to be an Eocene unit in the westernmost Qaidam basin [10,85,86], but this unit was recently demonstrated to be Early Oligocene (∼31 Ma) in age in its type section [87]. Magnetostratigraphic studies further show that deposition of the Lulehe conglomerate have not started until the Late Oligocene [52] or Early Miocene [70] in the Honggou section in the middle of the Qaidam basin, which is located *ca*. 120 km east of the type section. This situation clearly indicates that the Lulehe Formation is a diachronous unit, possibly resulting from eastward transgressive deposition. Conglomeratic facies associations of the unit represent alluvial–fluvial sedimentation at the edge of the basin. Expansion of the depositional area of the Qaidam basin is implied by the eastward onlap of Tertiary strata on geologic profiles (Fig. 7B) and isopach maps of different units [16,18]. Middle Tertiary successions are conformable in the western and central Qaidam basin, with the Oligocene Shangganchaigou Formation passing upward conformably into the Lower Miocene Xiayoushashan Formation. The two units are composed dominantly of lacustrine fine-grained siliciclastic and carbonate facies such as thin-bedded mudstone/siltstone and limestone/marls. The Paleogene is absent in the easternmost Qaidam basin or the Wulan basin where Miocene units lie unconformably over the Mesozoic and older rocks [88], similar to Tertiary stratigraphy of the Gonghe basin.

## SEDIMENTARY PROCESSES

### The West Qinling

Mid-Tertiary strata are well preserved in intermontane basins of the West Qinling (Figs 3 and 4). Oligocene units are comprised mostly of fine-grained facies associations deposited in lacustrine and meandering fluvial environments. Coarse-grained facies associations exist in some locales and result from fan-deltaic sedimentation due to high-rate extensional subsidence [62]. Paleosols are present at the uppermost parts of some Oligocene units, implicating development of exogenic surfaces at the end of the Paleogene. Lower Miocene successions usually commence with alluvial and braided fluvial debrite and coarse-grained sandstone, and unconformably overlie pre-Miocene rocks (Fig. 5A–D).

Complete Miocene successions perfectly crop out in the Guide basin south of the Laji Shan (Fig. 4). Alluvial and braided-river sedimentation marks the initiation of the Guide basin, as represented by basal debrite of the Guidemen Formation. Debrites are massive or thick-bedded, and consist dominantly of angular clasts of different lithologies such as metavolcanics, schist, quartzite, and argillite. Coarse-grained sandstone with planar and low-angle stratification becomes more abundant in the upper Guidemen Formation. Debrite and sandstone facies associations are interpreted as deposits of debris- and high-concentrated flows. Noteworthy is the rapid change from the Guidemen coarse-grained facies upward into green- and dark-colored fine-grained clastic facies and thin-bedded limestone, which make up the overlying Garang and Ashigong formations.

The Xunhua basin is located to the east of the Guide basin where the Oligocene unit is termed in the literature as either the Tala Formation or Xining Group. This unit consists of alluvial conglomeratic facies in the lower part and fluvial–lacustrine sandstone and mudrock in the middle and upper. In places, paleosol horizons are present in the uppermost part of the Tala Formation, which is then overlain disconformably by massive alluvial debrite and coarse-grained sandstone of the Guide Group. Fine-grained sandstone and mudrock faces become predominant in the middle Guide Group, and are deposited in lacustrine and deltaic environments. Braided river sedimentation took place in the north of the basin, as recorded by cross-bedded and parallel-stratified gravelly sandstone facies associations.

The Linxia and Xunhua basins are separated by the Jishi Shan, the southeastern extension of the Laji Shan (Fig. 4). Compared with its equivalent of the Xunhua basin, the Tala Formation is composed of meandering fluvial and lacustrine sandstones and siltstone, with conglomeratic beds being merely present at the base. The Lower Miocene Zhongzhuang Formation comprises conglomeratic facies in the lower part and fine-grained facies associations in the middle and upper. Our field observations and sedimentary analysis indicate that this unit exhibits a rapid transition from braided-river to meandering-river/lacustrine environments, similar to the Lower Miocene sequences in the Guide basin.

Middle Tertiary stratigraphy and sedimentation in the Tianshui basin northeast of the Linxia basin is also carefully studied [75]. The Oligocene Guyuan Group, less than 500 m thick, is made up primarily of massive conglomerate and coarse-grained sandstone, which are interpreted as debris-flow deposits in alluvial environments. The Miocene Ganquan Formation is comprised mostly of lacustrine siltstone and mudstone (Fig. 3), and rests disconformably on the Guyuan Group, with alluvial–fluvial conglomerate and sandstone being at the base [62].

**Figure 8.**
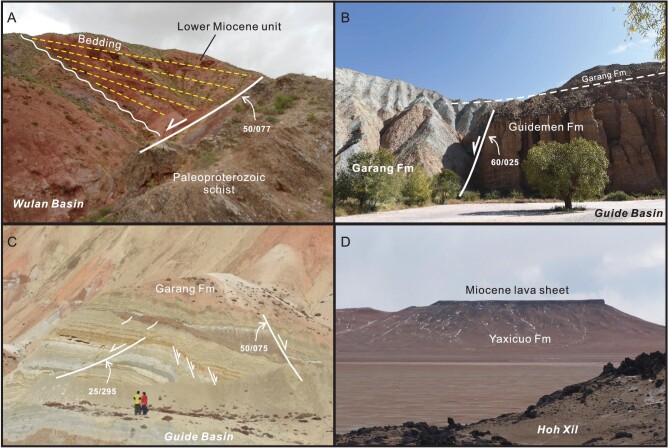
Field photos showing Early Miocene normal faults in the Wulan and Guide basins in the eastern Qaidam and West Qinling, and lava sheet in the Hoh Xil. (A) A normal fault controlling basal sedimentation of Lower Miocene unit in the Wulan basin, with a small depositional wedge thickening toward the fault. Width of view ∼20 m. (B) A normal fault separates the Guidemen from Garang units in the Guide basin, which is covered with the upper Garang fine-grained layer. Width of view ∼80 m. (C) Various-scale normal faults in the Garang sequence of the Guide basin. People for scale. (D) Early Miocene lava sheet in the distance, which overlies the Yaxicuo Formation and undergoes little deformation. View in the background 500 m wide.

It is worth noting that extension must have been active during the Early Miocene, as manifested by the presence of various-scale synsedimentary normal faults that either cut the unconformity beneath the Lower Miocene or occur in Lower Miocene successions. Figure 8A displays a normal fault controlling Lower Miocene sedimentation in the Wulan basin. The Wulan basin, located at present in the easternmost area of the Qaidam basin, was once connected with the Gonghe basin in the Early–Middle Miocene [89]. It is noticeable that the Miocene subsidence of the Gonghe basin was governed by normal faulting on its northern and southern borders, as inferred by geologic interpretation of a seismic profile [90]. In addition, normal faulting should have commenced at the beginning of the Early Miocene when the Guidemen and Garang successions were deposited. Normal faults cut the Guidemen conglomeritic sequences and controlled deposition of both the Guidemen alluvial conglomerate and Garang lacustrine fine-grained facies (Fig. 8B). Widespread presence of synsedimentary normal faults of various scales in the Garang succession (Fig. 8C) indicates continuation of normal faulting in the late Early Miocene. Normal faults are also documented in the eastern West Qinling, such the Tianshui basin [91,92]. Guo *et al*. [93] demonstrate that Miocene sedimentation in the Zhangxian basin should have taken place in an extensional setting based on detailed facies analysis of depositional successions. Miocene bimodal volcanic rocks in the West Qinling are also consistent with an extensional tectonic setting [94]. Extension might have affected the West Qinling until the Pliocene when contractional and transpressional deformation became prevailing [95].

### The Hoh Xil

The Late Eocene–Oligocene Yaxicuo Formation is made up primarily of fine-grained siliciclastic rocks, including thin- to medium-bedded sandstone, siltstone, and mudstone. Red-colored mudrock and sandstone typify the middle and upper parts of the formation, and gypsum layers are particularly common in the uppermost. Conglomerate and coarse-grained sandstone facies are present at the base. The fine-grained siliciclastic facies associations are interpreted as the result of coastal to shallow lacustrine sedimentation, whereas the basal conglomeratic facies formed in fluvial and deltaic environments [78,96–98].

The Wudaoliang Formation begins with conglomeratic facies, which pass upward to thin-bedded mudrock, bioclastic limestone, marl, and dolostone. Stromatolites of different morphology are commonplace, and can build up huge reefs or domes up to 80 m thick in some locales [99]. This unit is less than 400 m thick, varying from 100 to 350 m. Abundant ostracode fauna are reported, such as *Leucocythere* sp. and *Microlimnocythere* sp., *Ilyocypris* sp., *Eucypris* sp., and *Candona* sp. [82]. The sedimentary facies associations and ostracode fauna indicate that the Wudaoliang succession was deposited in lacustrine environments [82,97]. Broad distribution of fine-grained clastic and carbonate facies and the lack of coarse-grained facies are consistent with the inference of a vast lake in the Hoh Xil during the Early Miocene [82].

Also important is the occurrence of mid-Tertiary normal faults in northern Tibet [100]. Wu *et al*. [101] made a detailed investigation of the Wenquan basin that is bounded on the west by a normal fault, and demonstrated ∼18 Ma commencement of normal faulting. Blisniuk *et al*. [102] also documented an ∼13.5 Ma normal fault system that controlled the Shuanghu rift basin just south of the Wenquan basin. Unfortunately, there are no reports of synsedimentary normal faults in Miocene successions in the Hoh Xil in the literature although Pliocene–Quaternary extensional basins widely develop [80].

### The Qaidam basin

Mid-Tertiary sedimentary processes of the Qaidam basin have been extensively studied [42,86,103]. The Oligocene–Lower Miocene successions or the Shangganchaigou–Xiayoushashan successions are continuous, contrasting with the mid-Tertiary successions in the West Qinling and Hoh Xil. The successions are dominated by mudstone, siltstone, and carbonate rock, and there also occur stromatolite and algal reefs [104–106]. Fluvial systems mainly develop along the northern margin of the Qaidam basin. Existence of limestone, stromatolite, and algal reefs, in conjunction with the predominance of fine-grained clastic facies associations, hints at expansion of a vast lake during this period. Fluvial and alluvial systems began propagating into the basin from the surrounding structural belts in the Late Miocene, such as the East Kunlun [103], Altyn Tagh [107] and Qilian Shan [42], leading to considerable shrinkage of lake area and disappearance of carbonate deposition.

## MIOCENE VOLCANISM

**Figure 9.**
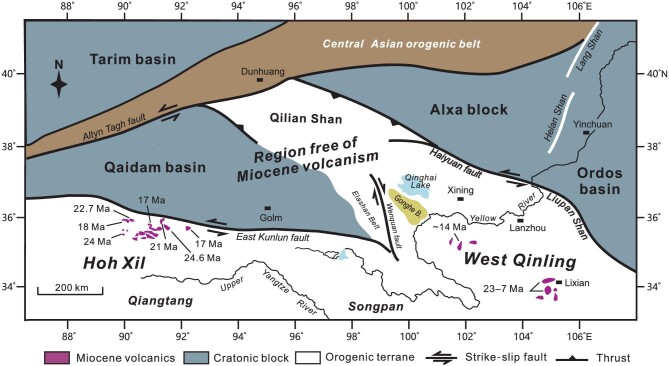
Diagram showing distribution of Miocene volcanics in NE Tibet. Note that volcanic rocks are distributed in the Hoh Xil and West Qinling but absent in the Qaidam basin, Qilian Shan and Alxa block. Geochronologic data of volcanics are from Refs [24,26,28,131,132].

Miocene volcanic rocks are distributed in the Hoh Xil and West Qinling (Fig. 9), and their petrology, geochemistry, and petrogenesis have been well studied [25,26,108–114]. Volcanic eruptions in the Qiangtang took place mainly in the Early–Middle Eocene, and then diminished [24,115–118]. Volcanism migrated northward and became quite active in the Hoh Xil and West Qinling during the Miocene (Fig. 9), as manifested by diverse volcanic lava dated at ∼23–7 Ma [26,28,113].

Mid-Tertiary volcanoes come in all sizes and shapes in NE Tibet, expressing themselves as positive landforms like lava sheets (Fig. 8D), domes and pyroclastic cones, ranging from 30 to 150 m in thickness [28,119,120]. Some lava sheets cover an area up to 200 km^2^ [121]. Volcanic rocks overlie sedimentary successions of different ages, with the youngest underlying strata being the Yaxicuo Formation. Volcanics are compositionally variable, including massive trachyte, trachyandesite, rhyolite, volcanic breccia and pyroclastics [28]. Intermediate rocks are basically potassium-rich adakitic volcanics and attributed to lower-crustal melting [26]. Peraluminous rhyolites, dated at 14–9 Ma, are associated with K-rich adakitic rocks, and ascribed to middle crustal melting [122]. It is particularly worth noting the occurrence of 16 Ma olivine leucitites in the Hoh Xil [26,28], which display very low SiO_2_ and high MgO contents, and are enriched in large ion lithophile elements [123]. These geochemical features indicate that olivine leucitites originate from low-degree partial melting of the lithospheric mantle [123]. Potassic–ultrapotassic volcanics are commonly thought of as the products of partial melting of the enriched lithospheric mantle at high temperatures [109,111]. Coexisting mafic rocks further indicate the contribution of partial melting of the lithospheric mantle to mid-Tertiary volcanism [124].

**Figure 10.**
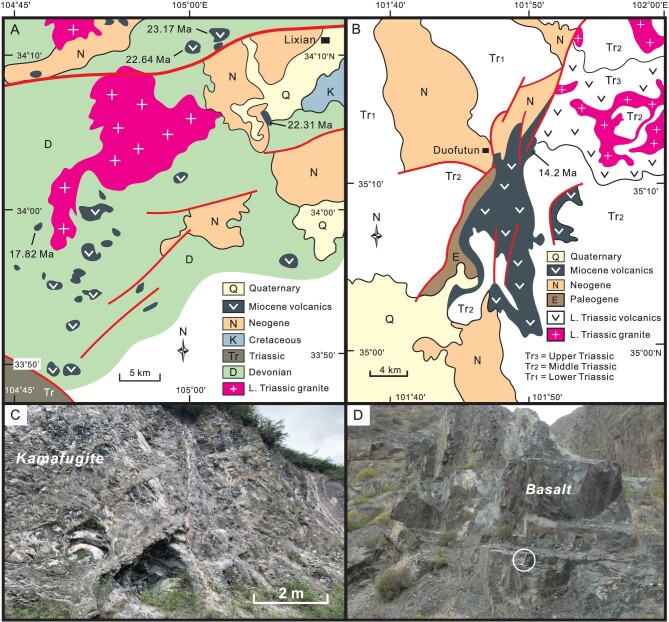
Diagram showing detailed distribution and outcrops of Miocene volcanic rocks in the West Qinling. (A) Volcanic rocks in the eastern West Qinling include kamafugite and minor felsic volcanics. Isotopic ages are from Ref. [113]. (B) Basalts in the middle West Qinling. Isotopic age is from Ref. [114]. (C) Kamafugite in the Lixian area, eastern West Qinling. (D) Basalts in the Tongren area, middle West Qinling. Hammer circled for scale.

Miocene lava in the eastern West Qinling is distributed as tens of individual outcrops usually <1 km^2^ (Fig. 10). Pyroclastic rocks occur as interlayers in volcanic lava successions. Volcanics are predominantly mafic and alkaline, and characterized by very high Na_2_O/K_2_O ratios and enrichment of Sr–Nd–Pb isotopic compositions. Timing of volcanic eruptions is constrained by phlogopite ^40^Ar/^39^Ar and zircon U-Pb ages from ∼23 to 7 Ma [113,114]. Kamafugite suites are typical of alkaline volcanic rocks in the eastern West Qinling (Fig. 10C), and accompanied by igneous carbonatite [125]. Porphyritic texture characterizes the kamafugites that contain abundant carbonatite inclusions [110,125], xenoliths of pyroxenite, dunite and harzburgite [25], and numerous phenocrysts of olivine, clinopyroxene and nepheline [25,126]. Miocene alkaline mafic and ultramafic volcanics in the middle part of the West Qinling express themselves as alkaline basalt and contain minor olivine crystals and carbonatite inclusions [125]. Basalts in the Duofutun area yield a zircon U-Pb age of ∼14 Ma (Fig. 10D) [114], and their geochemical characteristics implicate their derivation from partial melting of the carbonated mantle lithosphere [125].

No Miocene volcanics occur in the western West Qinling (Fig. 9). It is, however, interesting to note that hot dry rocks are widely reported in the Gonghe basin, and serve as important geothermal resources [127, 128]. Drilling reveals that hot dry rocks possess high temperatures up to 214°C at depths from 3700 to 4610 m, indicating a very high geothermal gradient (up to 14°C/100 m) and high terrestrial heat flows (up to 109.6 mW/m^2^) in the crust of the Gonghe basin [127,128]. Geophysical explorations attest to the existence of low-velocity zones (LVZs) at depths from 20–40 km, with calculated temperatures ranging from 680–760°C in the lower crust [128]. Plausibly, partial melting might have been happening in the mid-lower crust [129]. All these phenomena are suggestive of a deep-seated heat source beneath the Gonghe basin.

Broad distribution of diverse volcanic rocks and hot dry rocks collectively indicates thermal disturbance in the lithosphere of NE Tibet during the mid-Tertiary, and partial melting occurred in both the crust and lithospheric mantle. Crustal melting is indicated by rhyolite and K-rich adakitic rocks [26,122], whereas mantle sources of volcanics are sustained by the presence of peridotitic xenoliths in kamafugites in the West Qinling [25,27,113] and olivine leucitites and mafic–ultramafic rocks in the Hoh Xil [28,123,130]. It is estimated that potassic volcanics formed at 900–1200°C [26,111,131] and the lithospheric melting must have taken place at high temperatures up to 1400°C [132]. No mid-Tertiary volcanics have been reported in the Qaidam basin and Qilian Shan, in striking contrast to the vigorous volcanism in the adjacent Hoh Xil and West Qinling.

## DRIVERS FOR MID-TERTIARY UPLIFT OF NE TIBET

### Previous models

Many studies have been focusing on the age of inception of crustal shortening in NE Tibet. It is assumed that the India–Asia collision might have immediately affected the NE Tibetan Plateau, creating compressional basins in the Hoh Xil [29,78,79] and West Qinling [12,17,41]. Eocene–Oligocene conglomeratic sedimentation in the Qaidam basin is also ascribed to a far-field effect of the India–Asia collision [10,43]. Rapid Late Oligocene–Early Miocene exhumation of structural belts in the NE Tibetan Plateau, as recorded by low-temperature thermochronologic data, is considered to be the result of crustal shortening [22,45,51]. However, some tectonic explanations are inconsistent with our geologic observations.

Paleogene successions in the West Qinling were thought of as infill of compressional basins, with basin subsidence resulting from tectonic load applied by surrounding thrust sheets [12,41]. However, widely-observed synsedimentary normal faults of various scales in Paleogene successions hint at crustal stretching rather than shortening [62,133]. In addition, it is often taken for granted that conglomeratic sedimentation is indicative of shortening-related uplift of adjacent structural belts, and accordingly the Eocene–Oligocene Lulehe conglomerate in the Qaidam basin is frequently invoked to argue for activation of thrusting and uplifting of the Qilian Shan on the north. However, broad distribution and textural/petrologic maturation of the Lulehe conglomerate are at odds with the interpretation of rapid proximal subsidence and sedimentation of flexural basins.

There is also meager geological evidence bearing out Early Miocene crustal shortening. The view of Early Miocene crustal contraction in the northern Tibetan Plateau relies mainly upon exhumation history of structural belts, such as the East Kunlun [19,45], Qilian Shan [20] and Laji Shan [21], with cooling ages being utilized to constrain the initiation and chronicle of contraction-related uplifting. Unfortunately, few geologic observations attest to Early Miocene thrust faulting. Growth strata in well-dated Neogene successions show that folding and thrusting commenced ∼15 Ma and have persisted since then, as confirmed by seismic profiles in the Qaidam basin [16,18,46] and field observations in intermontane basins [53,54,134]. A dramatic change in sediment feeding systems, together with synchronous thrusting, also consistently points to ∼15 Ma commencement of rapid uplifting of structural belts in the NE Tibetan Plateau, such as the Qilian Shan [49,65], Jishi Shan [135], and intrabasinal thrust belts like the Luliang Shan in the northern Qaidam basin [64]. Strike-slip faulting along the edges of the NE Tibetan Plateau is also demonstrated to initiate at ∼15 Ma, such as the Altyn Tagh fault [48,136], East Kunlun fault [19,137] and Haiyuan fault [19,47]. Strike-slip faulting within the NE Tibetan Plateau began ∼10 Ma, such as the Elashan and Riyue Shan right-slip faults [57]. Moreover, Early Miocene prevalence of fine-grained clastic and carbonate sedimentation in the Qaidam basin and the West Qinling also negate the assumption of strong crustal contraction during that period of time. Active thrusting and uplifting would produce lots of coarse-grained clastics that rapidly propagate into basins and suppress carbonate sedimentation.

It is important to note another fact that crustal shortening in the northern Qiangtang and Hoh Xil had ceased by ∼27 Ma [79,81], and this view is verified by the observation of flat-lying Lower Miocene Wudaoliang Formation and lava sheets (Fig. 8D). It follows that the focused rock uplift of structural belts in the NE Tibetan Plateau is hardly ascribed to shortening as a result of tectonic push caused by the Asia–India collision. Instead, it is plausible that Late Oligocene–Early Miocene regional surface uplift, including the structural belts, arose from vertical crustal motion. This inference is buttressed by the occurrence of widespread disconformity separating Oligocene from Miocene units in both intermontane basins (Fig. 3) and structural belts such as the Laji Shan in the West Qinling (Fig. 5A). Moreover, the Hoh Xil surface height is estimated to have been ∼1400–2900 m in the Early Miocene in light of leaf fossils (barberry) in the Wudaoliang Formation [138]. Given that shortening had come to an end before ∼27 Ma in the Hoh Xil, exogenic processes and peneplanation was likely attributed to vertical surface uplift driven possibly by deep thermomechanical processes. The Hoh Xil surface elevation is now at ∼5000 m above sea level, and thus it must have experienced high-rate uplift since ∼15 Ma after the termination of Wudaoliang lacustrine deposition.

A number of dynamic models have been advanced to account for the rising of the Tibetan Plateau, such as northward-migrating crustal shortening and thickening [1,11] and convective removal of the mantle lithosphere [139]. These models are favored in that they provide some explanations of spatiotemporal variations of crustal deformation, surface elevation, and volcanism in the Tibetan Plateau as a whole [8,9,29,140], and are thus invoked to reconstruct Tertiary tectonic evolution of the Hoh Xil [78,79]. It is noteworthy that Molnar et al. [139] developed the mantle removal model to account for the rough synchronicity of crustal uplift and volcanism in the Late Miocene. However, Cenozoic volcanism had already begun prior to the Late Miocene, and exhibited striking spatial migration with time [109]. The mantle removal model is thus not fit for the mid-Tertiary tectonic evolution of NE Tibet. Another popular model appeals to southward subduction of the Asian continent to explain tectonic development of the Qaidam basins, surrounding structural belts [11,141], and Miocene volcanism in the Hoh Xil [26,142]. Unfortunately, this model fails to reconcile with some conspicuous features of mid-Tertiary tectonics of NE Tibet, as explicated in this study. First, crustal shortening had terminated by ∼27 Ma in the Hoh Xil, and the West Qinling was basically in an extensional regime in the Early Miocene. These facts are at variance with the postulation that crustal shortening had continued migrating northwards. Second, the convective removal model predicts simultaneous volcanism in a broad area, but Tertiary volcanism displays marked northward shifting, with volcanic eruptions occurring in the Qiangtang during the Eocene and starting in the Hoh Xil and West Qinling during the Miocene. Also noticeable is the absence of Miocene volcanics in the Qaidam and Qilian Shan where volcanism should have taken place if convective removal of lithospheric root happened beneath the NE Tibetan Plateau. Third, observations of flat-lying and gently-dipping Lower Miocene and younger lava sheets in the Hoh Xil are in conflict with tectonic models that assume southward subduction of the Asian continent along the East Kunlun belt and predict strong shortening of the overriding Hoh Xil.

### A new mechanism

It remains a key challenge to develop a dynamic model that could exposit mid-Tertiary diverse tectonic processes and their relationships in NE Tibet. A feasible mechanism should at least take into account the new observational data provided in this study, such as widespread disconformity between Oligocene and Miocene strata, Early Miocene normal faulting, and concurrent volcanism. The temperospatial coincidence among vertical surface uplift, supracrustal stretching and volcanism should be no accident. Petrogenesis of volcanic rocks in the Hoh Xil and West Qinling provides essential clues for inferring the driving forces. Potassic–ultrapotassic and mafic–ultramafic volcanic rocks, together with the presence of peridotitic xenoliths and olivine crystals, suggest that thermomechanical processes in the mantle should have played a significant role controlling crustal deformation in NE Tibet [108,109,111,125,143]. The missing Miocene volcanics in the Qaidam basin, Qilian Shan, and Alxa block is also an unavoidable fact (Fig. 9).

The crustal and lithospheric mantle structures of the Hoh Xil and West Qinling prove to be quite different from those of the Qaidam and Alxa blocks. Magnetotelluric data reveal that the Hoh Xil has a conductive or low-resistivity upper mantle, indicative of significant partial melting [144]. This fact is consistent with seismic images that indicate a hot upper mantle beneath the Hoh Xil [145]. Similarly, the West Qinling exhibits weak and diffusive negative velocity gradients in the lithospheric mantle, also implying thermal anomalies or high temperature [32]. By contrast, the Qaidam and Alxa blocks behave as rigid cratonic domains with higher resistivity [144]. Also interesting is the marked change in lithosphere thickness across the boundaries between orogenic and cratonic domains. The Hoh Xil and West Qinling possess relatively thinner lithosphere thickness varying from 125 to135 km [34,146] although their crust is considerably thicker, up to 65 km [147,148]. By comparison, the lithosphere of cratonic blocks is much thicker, varying from 175 to 190 km in the Qaidam basin and up to 200 km in the Alxa block, respectively [34]. Ye *et al*. [33] provide a deep seismic reflection profile across the boundary between the West Qinling and Alxa block, which illustrates a conspicuous change in lithosphere thickness between the two distinct tectonic domains. Speculations of active southward underthrusting of the Qaidam lithosphere beneath the Hoh Xil and the North China lithosphere beneath the West Qinling depend on interpretations of the seismic images [33,145] or are inferred from geochemistry of Miocene volcanics [26,122,142,149]. The conceptual models, however, are called into question by other geophysical investigations such as differential P- and S-wave travel-time measurement [31] and receiver-function imaging [150]. Moreover, the models are also at odds with geologic observations that Miocene strata basically remain little deformed in both the Hoh Xil and West Qinling.

The Songpan terrane, situated on the south of the West Qinling and merging with the Hoh Xil to the west, is part of the NE Tibetan Plateau (Fig. 1). This terrane consists mostly of strongly deformed Triassic turbiditic successions and is intruded by the scattering granites of Late Triassic–Early Jurassic ages [151,152]. The Songpan shares similar tectonic and magmatic evolution with the Hoh Xil and West Qinling in the early Mesozoic [153], but differs from the two orogens in some aspects. First, Cenozoic volcanics are missing. Second, Cretaceous strata remain essentially undeformed. Third, Cenozoic deposits are basically lacking [154]. These distinct features imply that the Songpan terrane escaped both deep thermomechanical processes and crustal compression in the Cenozoic. It is shown that the Songpan underwent two-phase rapid cooling at ∼120 Ma and ∼80 Ma during the Cretaceous, respectively, which was then followed by extremely slow surface uplift in the Cenozoic [154]. Implicitly, the Songpan might have already attained its present mean elevation of ∼3500 m prior to the Cenozoic. Crustal thickness of this terrane is up to ∼63 km [147], and the thickening might have primarily resulted from early Mesozoic shortening. Also notable is the thinner lithosphere of the Songpan terrane, varying from ∼125–135 km [34].

Cenozoic asthenospheric mantle flow is inferred to have taken place under the Tibetan Plateau in response to the persistent India–Asia convergence [155,156]. The plausibility of northward asthenospheric flow is supported by spatiotemporal variation of Cenozoic volcanism [143], and also extrapolated by seismic heterogeneity and anisotropy in the upper mantle [157] and P-wave velocity perturbations [158]. A thick LVZ is imaged in the upper mantle of the Hoh Xil, reaching down to the top of the mantle transition zone from the Moho [159]. The LVZ exists mainly beneath the Hoh Xil, West Qinling, and Songpan terranes [31,160], but disappears beneath the Qaidam and Alxa blocks [161]. Presumably, the northward asthenospheric mantle flow is driven by continued hinge advance of the subducting Indian lithospheric plate, a kind of tectonic process suggested by Kapp and DeCelles [6].

**Figure 11.**
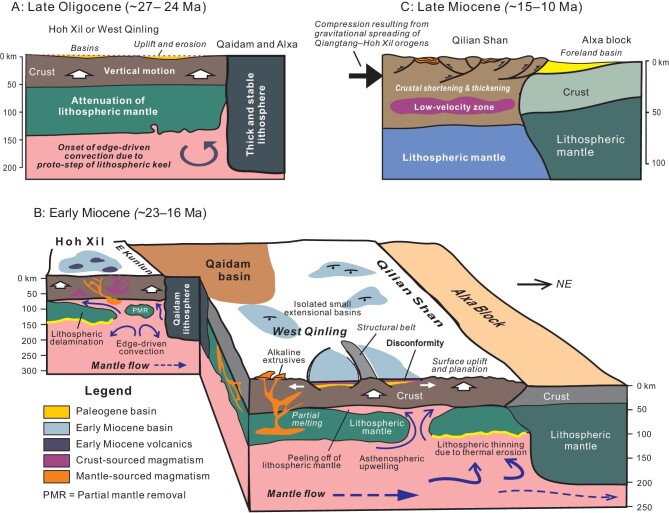
Tectonic model illustrating possible origin of vertical crustal motion and volcanism in the NE Tibetan Plateau in the mid-Tertiary. (A) Asthenospheric mantle flow caused by India-Asia collision was obstructed by the thicker cratonic lithosphere of the Qaidam and Alxa blocks, and the resulting edge-driven convection led to regional surface uplift of the West Qinling and Hoh Xil. (B) The edge convection brought about thinning and/or peeling of lithospheric mantle due to thermal erosion and asthenosphere uprising, and drove surface uplift of the West Qinling and Hoh Xil. Partial melting took place in both the lithospheric mantle and mid-lower crust due to heating of the asthenospheric materials which gave rise to volcanism sourced from the mid-lower crust and lithospheric mantle. Gravitational spreading of the elevated and heated orogenic crust resulted in supracrustal stretching and distributed small extensional basins. (C) Strong crustal shortening began affecting periphery of the NE Tibetan Plateau from the Late Miocene onward and led to thrust-related uplift of structural belts and basin inversion or shrinkage.

Based on a holistic treatment of geologic, geochemical and geophysical observations and data, we propose an edge-driven convection model that can satisfactorily account for regional surface uplift of the West Qinling and Hoh Xil as well as generation and spatial distribution of volcanic rocks in NE Tibet during the mid-Tertiary (Fig. 11). As described above, a marked step exists in lithosphere thickness between orogenic and cratonic domains, with the depth offset of the lithosphere–asthenosphere boundary (LAB) up to ∼45 km. Conceivably, the present-day variations in lithospheric thickness does not exactly reflect their original difference, for the lithosphere of the Hoh Xil and West Qinling must have been thinned as a result of thermal erosion or other thermomechanical processes (Fig. 11A and B). Nevertheless, cratonic blocks usually possess a considerably more stable and thicker lithosphere than adjacent orogenic belts [162]. This notion is well illustrated by the striking variation in lithosphere thickness between the Alxa block (>200 km) [163] and the Qilian Shan (∼170 km) [34], both of which have not been altered by Cenozoic deep thermomechanical processes. The discrepancy of LAB depth of the two domains should be even larger in consideration of the increase in crustal thickness of the Qilian Shan due to late Cenozoic shortening. If it is the case, lithospheric keels of the Qaidam and Alxa blocks could have served as barriers to impede the northward mantle flow, thereby inducing small-scale convection cells beneath the West Qinling and Hoh Xil since the Late Oligocene (Fig. 11A). The uprising asthenosphere heated the overlying lithosphere and led to partial melting of both the lithospheric mantle and mid-lower crust (Fig. 11B). Other thermomechanical processes might also be involved in the edge-driven convection, such as delamination and partial removal of the lithospheric mantle (Fig. 11B), especially beneath the Qiangtang–Hoh Xil that had experienced shortening just before the Late Oligocene [79]. K-rich adakitic and peraluminous rhyolitic volcanism resulted from partial melting of the mid-lower crust, whereas partial melting of the lithospheric mantle gave rise to mafic–ultramafic rocks and kamafugite suites. Gradual thermal erosion of the lithosphere by edge convection brought about vertical crustal uplift due to the replacement of metasomatosed mantle lithosphere by less dense asthenosphere (Fig. 11B), possibly in conjunction with magmatic inflation [130]. Upwelling of the asthenospheric hot materials is sustained by petrologic studies that infer very high temperatures from 800 to 1400°C in the crust and lithospheric mantle of the Qiangtang, Hoh Xil and West Qinling [111,131,132].

The edge convection exerted little effect on the lithosphere of the Qaidam basin, neither heating nor raising it. As a consequence, the Qaidam basin has remained a region of lower elevation (<1500 m) [107,164–166] and free from volcanism in the mid-Tertiary. The Qilian Shan was unaffected either by the edge convection owing to the defense of the Qaidam lithosphere keel on the south, thereby explaining the absence of Miocene volcanics in the Qilian Shan. Rapid uplift and exhumation of the Qilian Shan arose mainly from crustal shortening and thickening from ∼15 Ma [30,167]. The Songpan terrane appears to have also escaped the influence of edge-driven convection as a whole in that no volcanism and striking surface uplift happened there in the mid-Tertiary. The possibility is that the northward mantle flow had not been dammed up until it met the lithospheric keel of the Alxa block. The generated small-scale convection merely affected the West Qinling, and exerted little impact on the Songpan terrane. The focused Early Miocene exhumation of the East Kunlun and Laji Shan, as revealed by low-temperature thermochronologic data, should also be the consequence of convection-driven vertical crustal motion, presumably enhanced by normal faulting on the flanks of structural belts. Another outcome of vertical crustal uplift is supracrustal stretching in the West Qinling and Hoh Xil, possibly due to gravitational collapse (Fig. 11B). Supracrustal stretching created small-scale extensional basins filled with Lower Miocene successions, and was accompanied by volcanic eruptions. The convection-related vertical crustal uplift, therefore, provides a satisfactory explanation for the formation of the regional disconformity beneath Miocene strata and coeval volcanism in NE Tibet.

Crustal shortening has become predominant in the Qaidam basin and surrounding structural belts since Late Miocene time (Fig. 11C). It remains perplexing as to what caused the switch of tectonic regimes around 15 Ma. We conjecture that the change in tectonic settings might have resulted from gravitational collapse and spreading of the rising interior of the Tibetan Plateau. Our rationales are based on the following arguments. First, the Qiangtang and Hoh Xil are estimated to have been at a paleoelevation of <3000 m in the Early Miocene [138] and then reached an altitude of ∼4500 m in a short timespan from the Late Miocene to Pliocene [168], indicating a rapid uplift in the latest Neogene. Second, the Qaidam basin and surrounding structural belts had been regions of lower elevation of <1500 m prior to Late Miocene times, and then rapidly attained a high altitude of ∼3600 m in the Late Miocene [164]. Third, the Qiangtang and Hoh Xil are featured by the overthickened crust [149,169,170] and the presence of LVZs in the mid-lower crust [171]. The overthickened and heated orogens are weak and tend to collapse under internally-generated body force [1]. Finally, crustal stretching in the Qiangtang and Hoh Xil occurred simultaneously with marginal shortening of the East Kunlun and Qaidam basin in the Late Miocene. Taken together, a sharp elevation gradient could readily lead to gravitational collapse and spreading of hotter and thicker orogens toward regions of low elevation. Northward gravitational spreading of the Qiangtang and Hoh Xil is thus likely responsible for compressional force in marginal zones, thereby bringing about folding, thrusting and strike-slip faulting in the Qaidam basin and surrounding structural belts like the East Kunlun, Qilian Shan and Altyn Tagh belts. The West Qinling with ∼60-km-thick crust also underwent rapid elevation in the Late Miocene, as recorded by widespread planation surfaces [172], pollen assemblages [173], incision and reorganization of river systems [50], and supracrustal extension [92,93]. Noticeably, Late Miocene rapid uplifting of the West Qinling was coeval with left-slip faulting of the Haiyuan fault [47] and thrusting of the Longshoushan belt south of the Alxa block at low elevation [174]. The synchronicity of the two distinct tectonic activities implicates their genetic linkage, that is, gravitational spreading of the elevated West Qinling might have played a role in compressional deformations in its northern margin. Much more work is obviously required to unravel the relationships among diverse geologic processes in a holistic framework so as to advance a more feasible mechanism.

## CONCLUSIONS

Stratigraphic and sedimentologic investigations of mid-Tertiary successions demonstrate that the contact between Oligocene and Miocene strata is a regional unconformity in the West Qinling and Hoh Xil, but conformable in the Qaidam basin. This discordance manifests itself as a typical disconformity in the West Qinling, but as an angular unconformity in parts of the Hoh Xil, with Lower Miocene strata being nearly horizontal. Late Oligocene termination of shortening in the Hoh Xil and the formation of regional disconformity in the West Qinling indicate that the NE Tibetan Plateau experienced vertical surface uplift rather than crustal shortening in the mid-Tertiary. Widespread occurrences of synsedimentary normal faults in Lower Miocene succession undermines the long-held notion that Early Miocene intermontane basins in the West Qinling were created by crustal shortening. Miocene volcanism, sourced from both the crust and lithospheric mantle in the Hoh Xil and West Qinling, indicate the involvement of deep thermomechanical processes in mid-Tertiary tectonic processes. An edge-driven convection model is proposed that satisfactorily accounts for a number of prominent phenomena such as regional disconformity, synsedimentary normal faults and volcanic eruption in NE Tibet.
